# Investigating Chronotype and Sleep Quality in Psoriatic Patients: Results from an Observational, Web-Based Survey

**DOI:** 10.3390/jpm13111604

**Published:** 2023-11-13

**Authors:** Alessandro Borghi, Alfredo De Giorgi, Alberto Monti, Rosaria Cappadona, Roberto Manfredini, Monica Corazza

**Affiliations:** 1 Section of Dermatology and Infectious Diseases, Department of Medical Sciences, University of Ferrara, 44121 Ferrara, Italy; 2 Clinical Medicine Unit, Department of Medicine, Azienda Ospedaliero-Universitaria S. Anna, 44124 Ferrara, Italy; dgrlrd@unife.it; 3 University Center for Studies on Gender Medicine, Department of Medical Sciences, University of Ferrara, 44121 Ferrara, Italy

**Keywords:** psoriasis, sleep disturbance, chronotype, web survey, comorbidities

## Abstract

Background: Psoriasis is an inflammatory disease for which the implications and repercussions go far beyond the skin. Psoriasis patients suffer not only due to its skin manifestations and related symptoms but also because of comorbidities and a huge emotional impact. Objective: The objective of this study was to investigate chronotype and sleep quality in a group of Italian psoriatic patients. Materials and Methods: An observational, cross-sectional, web-based study was set up by the Dermatology and Clinical Medicine Sections of the Department of Medical Sciences, University of Ferrara, Italy. The web questionnaire was sent to an email list of an Italian association of psoriatic patients with the aim of recording their main demographic, social, historical, and clinical data. The survey included two questionnaires: the Morningness–Eveningness Questionnaire (MEQ) and the Pittsburg Sleep Quality Index (PSQI). Results: Two hundred and forty-three psoriatic patients (mean age 52.9 ± 12.8 yrs., 32.5% males and 67.5% females) filled out the questionnaire. A good 63.8% of them were affected with psoriasis for more than 10 years, 25.9% reported having a diffuse psoriasis, and 66.7% were on treatment at the time they completed the questionnaire. With reference to chronotype, the mean MEQ score was 55.2 ± 10.7; furthermore, 44% of the patients were “morning-oriented types”, M-types, or “larks”, 44.5% were “intermediate-types” or I-types, and 11.5% were “evening-oriented types”, E-types, or “owls”. No correlations were found between chronotype and psoriasis extension. Based on the PSQI results, 72.8% of the study population was judged to have a low sleep quality. Sleep disturbance was significantly related to female sex, living alone, and the presence of comorbidities. Conclusions: Sleep disturbance is very common in psoriatic patients, especially in those with comorbidities, in females, and in patients who live alone. The chronotype in psoriatic patients does not appear different when compared to the general population, nor does it seem to have any link with psoriasis severity.

## 1. Introduction

Psoriasis is a chronic inflammatory disease that primarily affects the skin. It has a multifactorial etiopathogenesis involving genetic susceptibility, immunological dysfunction, and environmental factors [1]. Psoriasis has a prevalence of about 2–3% worldwide, with relevant differences among regions, and it affects all ethnicities and ages [2]. In only the United States, according to an estimation based on the 2003–2004 National Health and Nutrition Examination Survey (NHANES) data, psoriasis affects 7.55 million US adults [3].

The abnormal release of pro-inflammatory cytokines, mainly polarized towards a Th17 phenotype, which underlies psoriatic cutaneous features, leads to a chronic systemic inflammatory state at the same time [4,5]. Thus, psoriasis can be considered a systemic inflammatory disorder that goes far beyond the skin [6,7]. In keeping with this, psoriatic patients suffer from comorbidities to a greater extent than the general population [1].

Accumulating evidence has shown the huge impact of psoriasis on patients’ well-being [8]. Its chronical course with recurrent flares, the skin disfigurement that can involve anatomical sites with a high social impact, and its distressing symptoms, including itching, are just a few of the factors that cause the strong disease-related burden and that impair quality of life.

Moreover, the bidirectional communication between the skin and the neural system is an increasingly recognized pathway that correlates mental stress and suffering with the exacerbation of inflammatory skin diseases [9]. This is also true for psoriasis, which represents a well-recognized model of the mutual etiopathogenetic influence between mental and skin disorders [10,11]. Chronic inflammatory background seems to be the most relevant common denominator linking psoriasis to its numerous and heterogeneous comorbidities, including those of the mental sphere [6].

With the intention of more deeply exploring the degree of suffering and distress related to psoriasis, several previous studies have addressed the correlation between psoriasis, sleep disturbance, and circadian rhythm.

The circadian system is regulated by a central clock, located in the suprachiasmatic nucleus, and it is entrained primarily by light/dark cycles via the retina. Modulating genes’ expression, it regulates organ and tissue physiology. On the other hand, genetic or environmental circadian dysregulation is involved in various diseases, including cancer, metabolic syndromes, cardiovascular disease, and immune-mediated disorders, like psoriasis [12,13,14]. The rhythmicity of psoriasis seems, to some extent, to be related to the dysregulation of the circadian fluctuation of angiogenesis and immunity mediators’ release [12,15].

Psoriasis-related itching has a cyclicity as well. It tends to occur or increase mainly in the evening and to worsen nocturnally [16]. Circadian dysfunctions, which, for example, affect cortisol levels and epidermal function, could explain the nocturnal worsening of pruritus [16,17] and thus account for sleep disruption [18].

In accordance with this, poor sleep quality has been found to be more common in psoriatic patients than in the general population [19,20,21,22,23].

Obstructive sleep apnoea and resting leg syndrome have been shown to be more common in psoriatic patients than in controls, and these conditions could further interfere with sleep quality [24,25,26,27,28].

But, the relationship between psoriasis and sleep disorders is even more complex, and a sort of vicious circle seems to exist. In other words, the relationship between sleep disturbance and psoriasis appears to be reciprocal. In fact, sleep deprivation in mouse models of psoriasis has been shown to increase levels of pro-inflammatory cytokines, which are a key physiopathological factor of psoriasis [29]. Thus, sleep deprivation could worsen psoriasis clinical features, thereby enhancing inflammation. This theory also seems to be indirectly supported by evidence on psoriatic patients [22,30].

However, to date, a precise correlation and a potential causal link between psoriasis and poor sleep are far from being defined. If sleep disturbance and psoriasis appear linked bi-directionally by biochemical and somatic factors, emotional variables certainly play an important role, too. Disease-related rumination, worry, anxiety, depression, intrusive thoughts, and, more generally, negatively toned emotional activity, which are quite common in psoriatic patients, seem to interfere with sleep quality [20,22].

Even though the circadian rhythm is the most recognized and relevant internal clock that regulates sleeping patterns and other biological processes, there is a large inter-individual variability in circadian rhythms because an individual circadian preference, the so-called chronotype, exists [31].

The chronotype defines the personal inclination of each individual to rest or carry out their activities throughout the day. Both biological and psychological parameters characterize a person’s chronotype. Based on chronotype, three main types of subjects can be identified: namely, morning types, evening types, and intermediate types [32].

A growing body of research indicates that chronotype is linked to different aspects of health, including unhealthy lifestyles, psychological health, sleep-related problems [33], diabetes mellitus [34], hepatic diseases [35], respiratory diseases [36], neurological diseases [37,38], and so on. Limited data are available on the potential impact of chronotype preferences on skin diseases [20,39,40].

The main objective of the present study was to specifically investigate chronotype and sleep quality in a sample population of Italian psoriatic patients in relation to their demographic, social, and clinical features and psoriasis treatments. In the speculative view of a personalized approach to every disease and relative treatment, the working hypothesis was that personal chronotype could be in relation to the severity of psoriasis and, hence, could negatively affect sleep quality.

## 2. Materials and Methods

### 2.1. Study Design and Patients

An observational cross-sectional study based on a web-based questionnaire was conducted by the Dermatology and Clinical Medicine Sections of the Department of Medical Sciences, University of Ferrara, Italy, between January and June 2023.

This novel, smart method is particularly appealing due to several advantages: it is relatively inexpensive, it is logistically flexible to implement, and it affords a high level of privacy and confidentiality when correctly implemented [41]. In fact, a web survey approach has recently been used for studies devoted to dermatologic topics, i.e., atopic dermatitis [42,43,44,45,46], and for other medical topics, as well [31,47,48,49,50].

The web-based questionnaire was sent to an email list of an Italian association of psoriatic patients named Associazione Psoriasici Italiani Amici della Fondazione Corazza (APIAFCO). APIAFCO is based in Bologna, northern Italy, but it is operating nationally and internationally, and it includes, overall, 2400 affiliates. The Corazza Foundation provides financial support for wide-ranging scientific research on psoriasis, psoriatic arthritis, and onco-dermatological diseases, both in Italy and abroad. Financing is addressed to valid research projects and to scholarships.

The psoriatic patients were invited to complete this web survey, which took approximately 15–20 min. Participants were fully informed on the survey’s cover page about the purpose of this study, their rights as participants, and the voluntary and confidential nature of participation, with all potential participants being informed that they were free to participate, refuse, or withdraw at any time. Anonymity was assured by using a progressive numerical code instead of participants’ names. No incentives were offered. Patients aged less than 18 years were excluded from this study. No further exclusion criteria were applied.

The questionnaire recorded the following data: (i) age, (ii) gender, (iii) educational attainment (a—primary school, b—middle school, c—high school, d—university degree or more), (iv) marital status (a—single, b—married/cohabiting, c—divorced, d—widowed), (v) employment (a—full- or part-time employed, b—retired, c—unemployed or looking for work, d—student, e—household), (vi) psoriasis duration, meant as the time between the patient-reported onset of its signs and/or symptoms and study inclusion, (vii) anatomical sites affected by psoriasis, (viii) previous and current treatments for psoriasis, and (ix) most relevant comorbidities. The survey also included the following questionnaires: (i) the Morningness–Eveningness Questionnaire (MEQ)—self-assessment version [32] and (ii) the Pittsburg Sleep Quality Index, validated Italian version [51].

The present study was conducted in agreement with the Declaration of Helsinki (1975, revised 2013). The responses were completely anonymous, so subjects could not be identified in any way. Moreover, each electronic file was deleted before analysis, which aimed at strengthening data anonymity and confidentiality. Ethics Committee approval was not required in these cases according to a national disposition by-law (G.U. n. 76, 31/03/2008).

### 2.2. Study Questionnaires

#### 2.2.1. Morningness–Eveningness Questionnaire, Self-Assessment Version (MEQ)

The Morningness–Eveningness Questionnaire (MEQ) is a reliable and validated measurement tool of chronotype [32]. It consists of 19 questions about personal daily sleep–wake habits and the preferred times of day for certain physical and mental activities. A 0–1 to 4–6 score, depending on the question, is assigned to each question, with an overall score ranging from 16 to 86. Higher MEQ scores reflect stronger preference for morningness (“morning-oriented types”, M-types) and lower scores reflect stronger preference for eveningness (“evening-oriented types”, E-types). Based on the overall score, five categories can be identified, namely: (i) definitely E-type (16–30), (ii) moderately E-type (31–41), (iii) neither type or Intermediate-type (I-type) (42–58), (iv) moderately M-type (59–69), and (v) definitely M-type (70–86). For ease of interpretation, we considered moderately E-type as E-type (16–41 points) and moderately M-type as M-type (59–86 points). Thus, we had three final subgroups, i.e., E-types (“owls”), I-types, and M-types (“larks”). The Italian version of the MEQ was included in the web-based questionnaire.

#### 2.2.2. Pittsburgh Sleep Quality Index (PSQI)

Sleep quality was assessed using the Italian version of the Pittsburgh Sleep Quality Index (PSQI) [51,52]. It is a self-rated questionnaire designed to evaluate the following 7 components of sleep over the past month: perceived sleep quality, sleep latency, sleep duration, sleep efficiency, sleep disturbance, use of sleep medications, and daytime dysfunction due to sleepiness. Each component yields a score ranging from 0 to 3, with 3 indicating the greatest dysfunction. A global score ranging from 0 to 21 is obtained, with higher scores indicating worse sleep quality. A global score ≥6 is suggestive of poor sleep quality.

Additionally, sex-based analyses were performed according to The Sex and Gender Equity in Research (SAGER) guideline [53].

### 2.3. Statistical Analysis

The survey was built on Google Forms and sent to the psoriatic patients affiliated with APIAFCO. Data were collected starting from 10 January 2023 to 30 June 2023. The statistical power of the sample to obtain statistically significant results was determined using a freely available online web platform. The authors considered the appropriate sample size for an adequate study power considering a confidence level of 99% and a confidence interval of 5% for a total of about 2400 psoriatic patients affiliated with APIAFCO. The analysis computed a representative sample size of 332 patients. The confidence interval, also called the margin of error, is the plus-or-minus figure usually reported in opinion poll results, whilst the confidence level suggests the level of security in excluding wrong answers.

The descriptive analysis included absolute numbers, percentages, and mean ± SD. Our outcome was the PSQI score, which was used to classify the population into individuals with a score <6 versus those with a score ≥6, the ≥6 score being the value related to poor sleep quality. The χ^2^ test, Student’s *t*-test, ANOVA test, and Kruskal–Wallis test were applied as appropriate. Logistic regression analysis was carried out in order to detect the factors associated with poor sleep quality (PSQI ≥ 6) by calculating the odds ratio (OR) and 95% confidence interval (CI).

In the sex model, the presence of at least one comorbidity and the tendency to live alone were the independent variables. Two-sided *p*-values < 0.05 were considered statistically significant. Statistical analysis was performed using IBM SPSS statistics (for Windows), version 26.0 (IBM Corp., Armonk, NY, USA).

## 3. Results

### 3.1. Study Patients

Over the 6 months from sending the questionnaire to the end of the recruitment period, a total of 243 psoriatic patients (mean age 52.9 ± 12.8 yrs.) responded and were included, of whom 79 (32.5%) were males and 164 (67.5%) females. The demographic and clinical characteristics of the study population are summarized in Table 1. The patients who responded to the questionnaire represented 10% of all those affiliated with the APIAFCO patient association.

The majority of the subjects included were married or partnered (65.9%) and employed (68.3%). More than half (59.7%) had comorbidities, even multiple.

Psoriasis had been diagnosed for more than 10 years in 63.8% of patients. About a quarter of the study subjects (25.9%) reported having a diffuse psoriasis, i.e., involving multiple skin districts, and, in variable percentages, psoriasis affected critical and/or difficult-to-treat skin areas, such as the head (68.7%), hands (35%), or genitals (20.6%). A large proportion of patients (66.7%) were already on treatment for psoriasis at the time of completing the questionnaire.

With reference to chronotype, the mean MEQ score was 55.2 ± 10.7. A substantial equivalence was found between “morning-oriented types”, M-types, or “larks” (44.0%) and “intermediate-types” or I-types (44.5%), while 11.5% of the population was an “evening-oriented type”, E-type, or “owl”.

Neither the mean MEQ score nor the distribution of the three different chronotypes were significantly different according to the extent of psoriasis based on the Kruskal–Wallis test or chi-squared test, respectively. The mean PSQI score was 7.72 ± 3.16.

### 3.2. Correlation between Quality of Sleep and Other Variables

The correlation between quality of sleep and the other variables recorded was investigated through univariate analysis (Table 2).

Considering the entire population as a whole and comparing the subjects (72.8%) with a PSQI score ≥6, indicating low sleep quality, with those with a score < 6 (27.2%), sleep disturbance was significantly correlated with female sex, marital status (in particular, living alone), and the presence of comorbidities. These associations were confirmed through multivariate analysis. No other significant correlations were found. In other words, psoriasis extent, involvement of critical skin districts, ongoing treatments, and chronotype were not correlated with sleep disturbance.

## 4. Discussion

The main objective of the present study, based on a web survey method, was to assess both sleep quality and chronotype in a large sample of Italian psoriatic patients. The population included was rather heterogeneous in terms of demographics, marital status, educational level, and extent of psoriasis (Table 1). There were patients (18.1%) with psoriasis limited to a single body area and patients with greater involvement, as well as patients with involvement of critical areas, like the hands, head, or genitals. A substantial rate of them (59.7%) had comorbidities, which is consistent with the recognized status of many psoriatic patients.

With specific reference to chronotype, the majority of the study subjects (88.5%) belonged to either the M-type or “larks” or I-type, with a numerical equivalence between these two categories. E-types, or “owls”, were decidedly a minority. This finding partly differs from that reported in a previous study, in which I-type subjects were the majority, being over 60% of the population [20]. Greater ethnic and geographic heterogeneity of the psoriatic patients included in that previous study could account for the difference in the distribution of the three chronotype categories.

The E-type has been associated with less healthy behaviours, e.g., smoking or lower physical activity, and it bears a higher risk for morbidity, including dysmetabolism, cardiovascular diseases, and depressive disorder [54,55,56], which are more common in psoriatic patients than in the general population [57,58]. In spite of this, in our population, the prevalence of the E-types was not higher than in the general population [59,60].

A noteworthy finding is that the three chronotypes, as well as the mean MEQ score, were not correlated with the extent of psoriasis. It is known that chronotype can influence personal habits, such as diet and lifestyle, skin biology [61], and various pathological conditions [33,62], as well; it can be influenced by some diseases [63]. Based on the findings of our study, chronotype does not appear to significantly influence the severity of psoriasis. This seems in disagreement with previous reports, which showed that night shift workers have a significant increase in their risk of developing both psoriasis and some comorbidities compared to people who are not involved in night shift work. Circadian system disruption in night shift workers probably underlies this observation through a modulation of the IL-23 receptor expressed in IL-17-producing cells [13,14]. Thus, the exact correlation between chronotype and psoriasis occurrence and severity remains unclear. It can be hypothesized that radical and/or chronic upheavals in the circadian cycle could have a negative effect on psoriasis, as in the case of workers subjected to work shifts. On the other hand, the simple predilection and predisposition to carry out activities during the morning rather than in the evening hours, which define the chronotype according to MEQ, would not be sufficient to determine significant variations in psoriasis expression.

With reference to the quality of sleep, which was evaluated by using the PSQI, a validated clinical tool for collecting both direct and indirect measurements of subjective sleep quality, we found a considerable rate of subjects (72.8%) with a poor sleep quality. This finding is quite in line with previous reports, which used the same measurement tool [19,20,64]. Comparing the prevalence of sleep disturbance among the included psoriatic patients with that recorded in the general population, which is estimated to range between 30 and 50% [65], impairment of sleep quality appears to be much more common in subjects affected with psoriasis. Therefore, our data confirm the sleep problems that have been previously revealed by a number of studies among psoriatic patients [23]. The correlation between psoriasis and sleep disorders seems rather complex, and multiple factors play a role in this regard. Pruritus has been indicated as the main cause of sleep disturbances in psoriatic patients [64,66], as in other skin disorders [67]. Due to its well-established responsibility, we did not address itching. Moreover, measurement and quantification of itching and of any psoriasis-related symptoms did not seem reliable when using a web collection method.

With reference to sex and gender, we observed that females had an increased risk of having sleep problems (Table 2). It is likely that, to some extent, these associations are independent of psoriasis, even though a recent study, which aimed to investigate sleep characteristics and clinical, demographic, and psychological factors associated with sleep disturbance in psoriasis, found that anxiety and depression levels were the strongest predictors of sleep impairment, followed by age, female sex, and pruritus [64]. In any case, sleep disturbance tends to be more common in female than in male subjects [68,69,70,71], regardless of concomitant chronic disorders.

Marital status deserves some consideration, as well. In our study, marital status (in particular, subjects living alone) had an impact on sleep problems. A study from our group, based on the available evidence in the literature, showed that being married (using the term married as “person with a partner”) was associated with lower risk factors and better health status, and males who were single generally had the poorest results [72]. A further meta-analysis with more than two million participants found that, compared with married participants, being unmarried (never married, divorced, or widowed) was associated with increased odds of cardiovascular disease (CVD), coronary heart disease (CHD), CHD death, and stroke death (OR 1.42, 1.16, 1.43, and 1.55, respectively) [73]. Again, data from a meta-analysis showed that being unmarried, divorced, or widowed is associated with a worse outcome in the heart failure population regarding either mortality or re-hospitalization rate (pooled OR = 1.52 and 1.80, respectively) [74]. Not only cardiovascular diseases, but also neoplasms, exhibit different outcomes depending on marital status. In fact, better outcomes have been observed in married patients when compared to unmarried (single, never-married, divorced/separated or widowed) in overall and cancer-specific survival. In particular, divorced/separated males were shown to be the most vulnerable group [75].

Last but not least, it is interesting to find a significant association between sleep disorders and comorbid patients. The presence of one or more concomitant diseases, in addition to psoriasis, represents a further element of sleep disturbance and may explain this finding. It remains to be defined whether comorbidities, and the suffering associated with them, are the cause of sleep disorders or whether, vice versa, insufficient sleep duration and poor sleep quality predispose to comorbidities, as it has been found [68].

We are aware of several limitations of this study. First, one limitation is the cross-sectional design based on data collected through a web survey; the sample could only be sized by internet users, and the final sample is only close to that theoretically calculated. Second, the MEQ score does not categorize for different ages [76]. Third, in regards to the evaluation of sleep, as this is a study based on a web survey, although we used validated questionnaires to investigate subjective sleep, a sleep diary remains the gold standard [77]. In fact, the subjective experience of sleep is a complex phenomenon with multiple components. A series of physiological sleep processes contribute to the morning perception of sleep, and more than 70% of the variance in the perception of sleep (based on a single observation per person) is not explained by overnight sleep-related physiological measures [78]. Fourth, regarding the test used and the possible sex/gender differences, sleep is considered to be an important determinant of mental and physical health, and a series of specific validated tests are available, each with pros and cons, in which sex and gender-specific differences are secondary. Across all questionnaires, for example, sleep quality was the strongest independent predictor of health, and, in particular, of mental health, and more so in females than in males [79]. We used the Pittsburgh Sleep Quality Index (PSQI), a well-known, validated, and reliable instrument for the measurement of the clinical construct of sleep quality, but a few studies have investigated possible sex and gender-specific differences. It has been shown that males and females may interpret what is meant by “overall sleep quality” in a different manner. For example, a question about perceived sleep quality was loaded with “sleep efficiency” and “sleep duration” in males and with “daytime dysfunction” and “sleep disturbances” in females [80]. Despite these last three points, the MEQ and PSQI questionnaires remain the most appropriate methods to be used for web-based studies, where easy handling, ease of response, and very limited time consumption are mandatory to warrant adherence. Fifth, this study did not include healthy controls, and the comparison with the general population was performed indirectly by using data available in the literature. Last, but not least, a dermatologist’s assessment of the severity, clinical subtype, and extent of psoriasis was lacking; psoriasis extent was reported by the patients themselves, and inaccuracies in this estimate cannot be excluded.

In spite of these limitations, the study results indicate that sleep disturbance is very common in psoriatic patients, especially in comorbid subjects, in females, and in patients who live alone. The results of this study are associative in nature, and an actual causal link is far from being proven. Contrary to our starting working hypothesis, at least for this sample of psoriatic patients, the chronotype does not appear particularly different compared to the general population and, in our experience, does not seem to have any relevance regarding the severity of psoriasis. However, personalized medicine—in other words, tailoring medical interventions to a particular patient—is a novel, intriguing field of research with already many applications in various fields of medicine, and it certainly deserves further in-depth studies, even in this dermatologic field.

## Figures and Tables

**Table 1:** Characteristics of the study population.

Patients, Total 243		
Chronotype		
	Morning-oriented type, “lark”, n. (%)	107 (44)
	Intermediate, n. (%)	108 (44.5)
	Evening-oriented type, “owl”, n. (%)	28 (11.5)
	MEQ (mean ± SD)	55.2 ± 10.7
Sleep quality		
	PSQI score (mean ± SD)	7.72 ± 3.16
	PSQI score ≥6, n. (%)	177 (72.8)
Demographics		
	Male, n. (%)	79 (32.5)
	Female, n. (%)	164 (67.5)
	Age (mean ± SD)	52.9 ± 12.8
Comorbidities		
	Rheumatologic, n. (%)	61 (25.1)
	Cardiovascular, n. (%)	42 (17.3)
	Cerebrovascular, n. (%)	6 (2.5)
	Pneumology, n. (%)	18 (7.4)
	Metabolic, n. (%)	13 (5.3)
	Gastrointestinal, n. (%)	7 (2.9)
	Oncological, n. (%)	7 (2.9)
	Renal, n. (%)	9 (3.7)
	Other, n. (%)	48 (19.8)
Number of comorbidities		
	None, n. (%)	98 (40.3)
	1 comorbidity, n. (%)	100 (41.2)
	2 comorbidities, n. (%)	28 (11.6)
	3 comorbidities, n. (%)	13 (5.3)
	4 comorbidities, n. (%)	4 (1.6)
	Number of comorbidities (mean ± SD)	0.87 ± 0.93
Marital status		
	Single, n. (%)	55 (22.6)
	Divorced—separated—widowed, n. (%)	28 (11.5)
	Married—cohabiting, n. (%)	160 (65.9)
	Living alone, n. (%)	83 (34.2)
Critical skin areas		
	Head and scalp, n. (%)	167 (68.7)
	Hands, n. (%)	85 (35)
	Head and scalp + hands, n. (%)	202 (83.1)
	Special areas (head and scalp, hands, genitalia), n. (%)	209 (86)
Psoriasis duration		
	<1 year, n. (%)	6 (2.5)
	2–5 years, n. (%)	44 (18.1)
	6–10 years, n. (%)	38 (15.6)
	>10 years, n. (%)	155 (63.8)
Treatments (previous and ongoing treatments)		
	Ongoing treatments, n. (%)	162 (66.7)
	Topicals, n. (%)	212 (87.2)
	Conventional systemic agents, n. (%)	77 (31.7)
	Biologicals or apremilast, n. (%)	62 (25.5)
	Phototherapy, n. (%)	76 (31.3)
	Topicals + Phototherapy, n. (%)	214 (88.1)
	Conventional systemic agents + Biologicals, n. (%)	107 (44)

MEQ, Morningness–Eveningness Questionnaire; PSQI, Pittsburgh Sleep Quality Index, SD, standard deviation.

**Table 2:** Univariate analysis of correlation between sleep quality and other study variables.

		PSQI < 6 (*n* = 66)	PSQI ≥ 6 (*n* = 177)	*p*
Chronotype				
	Morning-oriented type, “lark” (*n* = 107), n. (%)	31 (47)	76 (42.9)	0.265
	Intermediate (*n* = 108), n. (%)	31 (47)	77 (43.5)	
	Evening-oriented type, “owl” (*n* = 28), n. (%)	4 (6)	24 (13.6)	
	MEQ, mean ± SD	57.1±9	54.5±11.2	0.155
Demographics				
	Male (*n* = 79), n. (%)	31 (47)	48 (27.1)	**0.003**
	Female (*n* = 164), n. (%)	35 (53)	129 (72.9)	
	Age, mean ± SD, n. (%)	51.9 ± 12.9	53.2 ± 12.8	0.479
Comorbidities				
	Rheumatologic, n. (%)	11 (16.7)	50 (28.2)	0.064
	Cardiovascular, n. (%)	9 (13.6)	33 (18.6)	0.358
	Cerebrovascular, n. (%)	0	6 (3.4)	0.13
	Pneumology, n. (%)	3 (4.5)	15 (8.5)	0.298
	Metabolic, n. (%)	4 (6.1)	9 (5.1)	0.764
	Gastrointestinal, n. (%)	2 (3)	5 (2.8)	0.932
	Oncological, n. (%)	3 (4.5)	4 (2.3)	0.343
	Renal, n. (%)	2 (3)	7 (4)	0.734
	Other, n. (%)	8 (12.1)	40 (22.6)	0.068
Number of comorbidities				
	None (*n* = 98), n. (%)	36 (54.5)	62 (35)	**0.016**
	1 comorbidity (*n* = 100), n. (%)	22 (33.4)	78 (44.1)	
	2 comorbidities (*n* = 28), n. (%)	6 (9.1)	22 (12.4)	
	3 comorbidities (*n* = 13), n. (%)	0	13 (7.4)	
	4 comorbidities (*n* = 4), n. (%)	2 (3)	2 (1.1)	
	Number of comorbidities, mean ± SD	0.64 ± 0.89	0.95 ± 0.93	**0.006**
Marital status				
	Single (*n* = 55), n. (%)	12 (18.2)	43 (24.3)	**0.04**
	Divorced—separated—widowed (*n* = 28), n. (%)	3 (4.5)	25 (14.1)	
	Married—cohabiting (*n* = 160), n. (%)	51 (77.3)	109 (61.6)	
	Living alone (*n* = 83), n. (%)	15 (22.7)	68 (38.4)	**0.022**
Involved skin areas				
	Head and scalp, n. (%)	45 (68.2)	122 (68.9)	0.911
	Hands, n. (%)	21 (31.8)	64 (36.2)	0.528
	Head and scalp + hands, n. (%)	58 (87.9)	144 (81.4)	0.227
	Special areas (head and scalp, hands, genitalia), n. (%)	58 (87.9)	151 (85.3)	0.608
	1 area involved, n. (%)	11 (16.7)	33 (18.7)	0.935
	2–3 areas involved, n. (%)	34 (51.5)	88 (49.7)	
	4 or more areas involved, n. (%)	21 (31.8)	56 (31.6)	
Psoriasis duration				
	<1 year, n. (%)	1 (1.5)	5 (2.8)	0.815
	2–5 years, n. (%)	11 (16.7)	33 (18.7)	
	6–10 years, n. (%)	9 (13.6)	29 (16.4)	
	>10 years, n. (%)	45 (68.2)	110 (62.1)	
Treatments (previous and ongoing treatments)				
	Ongoing treatments, n. (%)	42 (63.6)	120 (67.8)	0.541
	Topicals, n. (%)	59 (89.4)	153 (86.4)	0.539
	Conventional systemic agents, n. (%)	18 (27.3)	59 (33.3)	0.366
	Biologicals or apremilast, n. (%)	15 (22.7)	47 (26.6)	0.543
	Phototherapy, n. (%)	19 (28.8)	57 (32.2)	0.609
	Topicals + Phototherapy, n. (%)	59 (89.4)	155 (87.6)	0.697
	Conventional systemic agents + Biologicals, n. (%)	25 (37.9)	82 (46.3)	0.238

MEQ, Morningness–Eveningness Questionnaire (MEQ); PSQI, Pittsburgh Sleep Quality Index; significant values are shown in bold.

## Data Availability

Data are contained within the article.
